# Tomatidine reduces Chikungunya virus progeny release by controlling viral protein expression

**DOI:** 10.1371/journal.pntd.0009916

**Published:** 2021-11-11

**Authors:** Berit Troost-Kind, Martijn J. van Hemert, Denise van de Pol, Heidi van der Ende-Metselaar, Andres Merits, Malte Borggrewe, Izabela A. Rodenhuis-Zybert, Jolanda M. Smit

**Affiliations:** 1 Department of Medical Microbiology and Infection Prevention, University of Groningen; University Medical Center Groningen; Groningen, the Netherlands; 2 Department of Medical Microbiology, Molecular Virology Laboratory, Leiden University Medical Center, Leiden, the Netherlands; 3 Institute of Technology, University of Tartu, Tartu, Estonia; 4 Department of Biomedical Sciences of Cells & Systems, Section Molecular Neurobiology, University of Groningen; University Medical Center Groningen; Groningen, the Netherlands; University of Glasgow, UNITED KINGDOM

## Abstract

Tomatidine, a natural steroidal alkaloid from unripe green tomatoes has been shown to exhibit many health benefits. We recently provided *in vitro* evidence that tomatidine reduces the infectivity of Dengue virus (DENV) and Chikungunya virus (CHIKV), two medically important arthropod-borne human infections for which no treatment options are available. We observed a potent antiviral effect with EC50 values of 0.82 μM for DENV-2 and 1.3 μM for CHIKV-LR. In this study, we investigated how tomatidine controls CHIKV infectivity. Using mass spectrometry, we identified that tomatidine induces the expression of p62, CD98, metallothionein and thioredoxin-related transmembrane protein 2 in Huh7 cells. The hits p62 and CD98 were validated, yet subsequent analysis revealed that they are not responsible for the observed antiviral effect. In parallel, we sought to identify at which step of the virus replication cycle tomatidine controls virus infectivity. A strong antiviral effect was seen when *in vitro* transcribed CHIKV RNA was transfected into Huh7 cells treated with tomatidine, thereby excluding a role for tomatidine during CHIKV cell entry. Subsequent determination of the number of intracellular viral RNA copies and viral protein expression levels during natural infection revealed that tomatidine reduces the RNA copy number and viral protein expression levels in infected cells. Once cells are infected, tomatidine is not able to interfere with active RNA replication yet it can reduce viral protein expression. Collectively, the results delineate that tomatidine controls viral protein expression to exert its antiviral activity. Lastly, sequential passaging of CHIKV in presence of tomatidine did not lead to viral resistance. Collectively, these results further emphasize the potential of tomatidine as an antiviral treatment towards CHIKV infection.

## Author summary

Chikungunya fever is a debilitating disease caused by the mosquito-borne Chikungunya virus. Over the past two decades the geographical spread of the virus and its mosquito vector has drastically increased thereby causing millions of infections. To date there is no antiviral drug and no vaccine available to treat/prevent Chikungunya virus infection. We recently showed that the natural steroidal alkaloid tomatidine has potent antiviral activity towards Chikungunya virus at submicromolar concentrations. In this study we dissected how tomatidine reduces the production of Chikungunya virus particles. We show that tomatidine lowers viral protein expression and we hypothesize that the effect of tomatidine on viral protein translation hampers the production of progeny viral RNA copies / number of infected cells thereby leading to a reduced production of secreted virus particles. Also, we show that Chikungunya virus does not readily become resistant to tomatidine. Collectively, we deciphered the mechanism by which tomatidine exerts antiviral activity to Chikungunya virus and our results strengthen the potential of tomatidine as an antiviral treatment strategy towards Chikungunya virus.

## Introduction

Chikungunya virus (CHIKV) is a re-emerging arbovirus transmitted to humans via the mosquito vectors *Aedes aegypti* and *Aedes albopictus* [1]. Until 2004 the virus was limited to Africa and Asia; however, following the recurrence of CHIKV in Kenya in 2005, CHIKV has globally expanded from the tropical zone to America and Europe causing millions of infections [2,3]. CHIKV typically causes an acute self-limiting febrile disease including symptoms such as fever, rash, nausea, fatigue, headache and myalgia [4]. In 30–40% of the cases, the disease progresses to a chronic form involving debilitating muscle and join pains which can persist for months to years after the initial infection [5].

Currently, there are no licensed antiviral treatments or vaccines available to combat CHIKV infection. Treatment is limited to administration of pain killers, antipyretics and non-steroidal anti-inflammatory drugs to alleviate the symptoms [6]. Antiviral therapies aim to reduce viral replication by directly targeting a viral protein (direct-acting antivirals) or by modulating host factors that are crucial for replication (host-directed antivirals) [7]. Previous *in vitro* CHIKV research identified both types of antivirals [7]. Direct-acting antivirals were shown to target structural (capsid, envelope E1 and E2, 6k) and non-structural proteins (nsP1, nsP2, nsP3 or nsP4) of CHIKV. Host-directed antivirals were found to target various host factors including kinases (e.g. CND3514 [8]), furin proteases (e.g. decanoyl-RVKR-chloromethyl [9]), HSP-90 (e.g. ganetespib [10]) endosomal acidification (e.g. chloroquine [11]), inosine monophosphate dehydrogenase (e.g. ribavirin [12,13]) and interferon (e.g. recombinant IFN-α [14]). Thus far, two antivirals have been tested in clinical trials: the virus cell entry inhibitor chloroquine and the inosine monophosphate dehydrogenase inhibitor ribavirin; however, without success [15,16]. This shows the need to follow-up more and identify new antiviral compounds.

Recently, we identified tomatidine, a natural steroidal alkaloid extracted from the stems and leaves of green tomatoes, as a potent inhibitor of different CHIKV and Dengue virus (DENV) strains *in vitro* [17,18]. Tomatidine was shown to interfere with CHIKV production in a variety of cell lines including, African green monkey Vero cells, human skin fibroblast HFF-1 cells, human osteosarcoma U2OS cells and in human hepatic Huh-7 cells thereby excluding a cell type specific effect [17]. Tomatidine is the aglycone metabolite of tomatine, which naturally protects the tomato plant from pathogens such as fungi, viruses, bacteria and insects [19,20]. Tomatidine has been shown to exert various health benefits including an anti-inflammatory, anti-metastatic, anti-atherosclerotic, anti-osteoporosis and antimicrobial activity [20–27]. Moreover, tomatidine was shown to protect from age-related muscle atrophy and ischemic neuronal injury [28,29]. To identify the mechanism of protection from muscle atrophy, an microarray analysis on mouse skeletal muscle cells was performed and subsequent gene set enrichment analysis revealed 7 gene sets significantly induced and 25 gene sets significantly suppressed in the presence of tomatidine [28]. Among the suppressed gene set was the transcription factor ATF4, which was identified as a key mediator of age-related muscle atrophy. We tested the importance of ATF4 in the antiviral effect of tomatidine towards DENV and revealed that although it might be involved it is not the main factor responsible for the antiviral effect [18]. Furthermore, in the context of its anti-atherosclerotic effect, tomatidine was shown to inhibit acyl-CoA:cholesterol acyl-transferase-1 and 2, which are enzymes involved in the formation of foam cells, a characteristic feature of atherosclerotic lesions [24]. A study on the anti-inflammatory properties of tomatidine demonstrated that it has the ability to inhibit the NF-kB and JNK pathway [21]. Thus, exposure of cells to tomatidine modulates various pathways within the cell.

Here, we analyzed the mechanism underlying the antiviral activity of tomatidine towards CHIKV in Huh7 cells from the host cell as well as the virus perspective. In our experimental set-up, tomatidine treatment had no major consequences on the host cell proteome and the validated proteins are not responsible for the observed antiviral effect. Subsequent analysis of the steps in the viral replication cycle suggests that tomatidine controls the expression of viral proteins thereby reducing virus progeny secretion. Furthermore, sequential passaging of CHIKV under tomatidine selection pressure revealed that viral resistance is not quickly obtained. In conclusion, these results further strengthen the potential of tomatidine as an antiviral compound towards CHIKV.

## Methods

### Chemicals

Tomatidine hydrochloride and ivermectin were purchased from Sigma Aldrich (St. Louis, Missouri, USA) and dissolved in absolute ethanol to a stock solution of 5 mM. Aliquots were stored at -20°C and were used for a maximum of 3 months. The final concentration of ethanol was below 0.1% in all experiments.

### Cell culture

The human hepatic cell line Huh7 (JCRB0403) was a kind gift from Tonya Colpitts (University of South Carolina). Cells were maintained in Dulbecco’s minimal essential medium (DMEM) Glutamax (Gibco, The Netherlands) supplemented with 10% FBS (Lonza, Basel, Switzerland) and 100 U/mL penicillin and 100 mg/mL streptomycin (PAA Laboratories, Pasching, Austria). The renal African Green monkey cell line Vero-WHO, was maintained in DMEM supplemented with 5% FBS, 100 U/mL penicillin and 100 mg/mL streptomycin. All cells were cultured at a temperature of 37°C with 5% CO_2_.

### Mass spectrometry

Huh7 cells were lysed using the RIPA lysis buffer system (Santa Cruz Biotechnology, Dallas, Texas, USA) and proteins were extracted according to the manufacturer’s instructions. Samples were loaded on a 4–12% pre-cast NuPAGE gel (Invitrogen, Carlsbad, California, USA), and briefly ran into the gel, without separation. The gel was stained with Coomassie dye R-250 (Thermo Fisher Scientific, Waltham, Massachusetts, USA) followed by destaining with ultrapure water. The protein blob was excised as a single band and destained with 70% 50 mM NH_4_HCO_3_ and 30% acetonitrile. Reduction was performed using 10 mM DTT dissolved in 50 mM NH_4_HCO_3_ for 30 min at 55°C. Samples were alkylated using 55 mM iodoacetamide in 50 mM NH_4_HCO_3_ for 30 min at room temperature. Subsequently, samples were washed for 10 min with 50 mM NH_4_HCO_3_ and for 30 min with 100% acetonitrile. The gel pieces were dried for 15 min at 55°C. Tryptic digestion was performed with sequencing-grade modified trypsin (Promega, Madison, Wisconsin, USA, 10 ng/ml in 50 mM NH_4_HCO_3_) with overnight incubation at 37°C. Peptides were extracted using 5% formic acid followed by a second elution with 5% formic acid in 75% acetonitrile. Samples were dried in a SpeedVac centrifuge and dissolved in 5% formic acid for injection on an Ultimate 3000 nanoLC system (Dionex, Sunnyvale, California, USA) interfaced online with a Q Exactive Plus mass spectrometer (Thermo Fisher Scientific). Samples were loaded onto a 5 mm × 300 μm i.d. C18 PepMAP100 trapping column (Dionex) in 2% acetonitrile in water with 0.1% formic acid at 20 μL/min. Peptides were separated on a 50 cm × 75 μm i.d. C18 PepMAP100 nanocolumn (Dionex). The following mobile phase gradient was delivered at the flow rate of 300 nL/min: 2%-50% of solvent B in 90 min; 50%–90% B in 1 min; 90% B during 13 min, and back to 2% B in 1 min (held for 15 min). Solvent A was 100 water with 0.1% formic acid, and solvent B was acetonitrile with 0.1% formic acid. Peptides were infused into the mass spectrometer using a stainless-steel emitter at a spray voltage was 1.8 kV with no sheath and auxiliary gas flow; the ion transfer tube temperature was 275°C. The mass spectrometer was operated in data-dependent mode (DDA) with MS/MS fragmentation of the top 12 precursor ions in each cycle. A cycle consisted of a survey scan of *m/z* 400–1800 at 70000 resolution and MS/MS scans at 17500 resolution. Singly charged ions were excluded from MS/MS experiments and precursor ions were dynamically excluded for 30 s. Proteins with no intensity in at least one sample were filtered. EdgeR R-package (v3.24.3) was used for normalization (trimmed mean of M values; TMM) and differential protein expression analysis (glmQLFTest) [30]. Proteins were regarded differentially expressed with a false discovery rate (FDR) lower than 0.1. The mass spectrometry proteomics data have been deposited to the ProteomeXchange consortium via the PRIDE partner repository with the dataset identifier PXD028693.

### Virus stocks and titration

The La Reunion CHIKV isolate (LR2006 OPY1, East/Central/South African genotype was rescued from the corresponding infectious cDNA clone and produced on Vero-WHO cells as described by Troost *et al* [17]. The infectious virus titer was determined via plaque assay on Vero-WHO cells as number of plaque forming units (PFU) as described before [17]. The intracellular viral genome copy number was determined via Q-RT-PCR. The primers are described in Troost *et al* and bind within the CHIKV E1 region [17]. Cellular RNA was isolated using the RNeasy kit (Qiagen, Hilden, Germany) according to the manufacturer’s protocol. The cDNA production and Q-RT-PCR were performed as described previously [17,31].

### Infection experiments

Huh7 cells were infected with CHIKV at the multiplicity of infection (MOI) 1 in virus resistance studies or MOI 10 in mode-of-action experiments. In mode-of action experiments, a high MOI was used to increase the sensitivity of the experiment. Tomatidine (10 μM) or ivermectin (7 μM) treatment was added at the time of infection unless otherwise indicated. As a vehicle control the equivalent volume of the EtOH solvent was used. The virus inoculum was removed 2 hours post-infection (hpi), cells were washed three times with plain medium and fresh compound-containing medium was added to the cells unless indicated otherwise.

### Protein extraction and western blot

Huh7 cells were lysed via the RIPA lysis buffer system (Santa Cruz Biotechnology) and protein was extracted according to the manufacturer’s instructions. The protein concentration was determined using the Bradford assay (Expedeon, Swavesey, UK). Sample preparation and western blot analysis were performed as described before [18]. The primary antibodies were diluted as follows: GAPDH (Abcam, Cambridge, UK) 1:10,000, vinculin (Sigma-Aldrich) 1:500, CD98 (Sino Biological, Peking, China) and p62 (Progen, Heidelberg, Germany) 1:500, thioredoxin-related transmembrane protein 2 (Thermo Fisher Scientific) 1:125, and nsP2 (Abgenex, Bhubaneswar, Odisha, India), E1 (kindly provided by Gorben Pijman, Wageningen University) and capsid (Native antigen company, Kidlington, UK) 1:1,000.

Dilutions of the antibodies were prepared in TBS-Tween with 0.1% of sodium azide and either 5% BSA or 5% milk. Secondary antibodies conjugated with HRP anti-mouse or anti-rabbit (Thermo Fisher Scientific) were used according to the manufacturer’s recommendations. Pierce ECL western blotting substrate (Thermo Fisher Scientific) or Super Signal West FEMTO (Thermo Fisher Scientific) was used for detection by chemiluminescence using LAS-4000 mini camera system (GE Healthcare, Chicago, Illinois, USA). Analysis was performed with the Image QuantTL software (GE Healthcare). The band intensity of each protein was normalized to that of the loading control (GAPDH or vinculin) and expressed as the fold-change over EtOH-treated cells.

### Transfection of siRNAs

The siRNAs p62 #1(ID: J-010230-05-0005), P62 #4(ID: J-010230-08-0002), CD98 (SLC3A2, ID: L-003542-00-0005) or non-targeting siRNA (siScramble, ID: D-001810-10) were purchased from Dharmacon (Horizon, Lafayette, Colorado, USA). Reverse transfection of Huh7 cells was performed using Lipofectamine RNAiMAX (Invitrogen) with a final concentration of 20 nM for the siRNAs according to the manufacturer’s protocol. Cells were seeded in a 12-well plate at a density of 7*10^4^ cells per well. At 48 h post-transfection, cells were infected with CHIKV at MOI 10 and treated with 10 μM tomatidine or the equivalent volume of EtOH. Supernatants were collected at 9 hpi and used to titrate the produced virus using plaque assay. Cells were harvested and subjected to WB analysis to determine the expression of p62 and CD98 proteins.

### Electroporation study

*In vitro* transcribed RNA was produced from the infectious clone CHIK-LR [32]. The RNA was electroporated into Huh7 cells (260 V and 950 μF) via the Gene Pulser Xcell system (Bio-Rad, Hercules, California, USA). Cells were counted and seeded into a 12-well plate containing medium supplemented with 10 μM tomatidine or the equivalent volume of EtOH at a density of 0.3*10^6^ cells per well. Supernatants were collected 16 h post-electroporation and the infectious virus titer was determined by plaque assay.

### Replication study

The *trans*-replicase system of CHIKV consisted of two plasmids one encoding for the CHIKV-LR replicase and the other for the truncated CHIKV RNA template containing a sequence encoding for a tomato fluorescent marker protein under the control of the CHIKV subgenomic promotor. As a control, a plasmid expressing an inactive form of the CHIKV replicase was used. Inactivity was due to a mutation in the catalytic Gly-Asp-Asp (GDD) motif of nsP4 to Gly-Ala-Ala (GAA) [33]. Huh7 cells were seeded in a 24 well-plate at a density of 0.6x10^5^ cells/well. At 24 h post-seeding, cells were co-transfected with 0.25 μg of both plasmids using Lipofectamine LTX reagent (Invitrogen) according to manufacturer’s protocol. At 1 h post-transfection, 10 μM tomatidine, 7 μM ivermectin or the equivalent volume of EtOH was added to the cells. Cells were collected at 24 hpi, fixed and analyzed for the expression of the tomato fluorescent protein using flow cytometry. The expression of the marker indicates active replication in cells transfected with of this *trans*-replicase system.

### In-gel hybridization

Huh7 cells were infected with CHIKV at MOI 10, as described above. Cells were harvested at 6 hpi and cellular RNA was isolated using the RNeasy kit (Qiagen). Denaturating formaldehyde gel electrophoresis was performed as previously described [34]. RNA was detected via ^32^P-labelled oligonucleotides via in-gel hybridization. Hereto, the ^32^P-labelled probe CHIKV Hyb4 (5’TGTGGGTTCGGAGAATCGTGGAAGAGTT-3′) and as control the 18S ribosomal RNA probe (5′-ATGCCCCCGGCCGTCCCTCT-3′) were used, as described previously [34]. For detection of the ^32^P-labelled RNA, dried gels were exposed to a Phosphor Imaginer screen and then scanned via a Tryphoon 9410 imager (GE Healthcare).

### Time-trace and time-chase experiments

In the time-trace experiments, Huh7 cells were infected with CHIKV at MOI 10 and treated with 10 μM tomatidine, as described above. Cellular RNA was extracted at 2, 4, 6 or 8 hpi. For the time-chase experiments, tomatidine was added at 6 hpi and cellular RNA was isolated at 8 and 10 hpi. Intracellular RNA was isolated with the RNeasy kit (Qiagen) and Q-RT-PCR was performed to determine the intracellular RNA copies [17].

### Resistance study

We used two commonly used approaches to evaluate the possible development of virus resistance to tomatidine. In the first approach, the virus was initially infected at MOI 1 and then continuously passaged using a 1:100 dilution of the virus-containing supernatant on Huh7 cells in the presence or absence of tomatidine. With each passage, the tomatidine concentration was increased from 2 μM to 4, 6, 7, 8, 9 and 10 μM. After reaching 10 μM, the remaining passages (passage 8 to 15) were performed in the presence of 10 μM tomatidine. During infection, the virus inoculum was not removed. The infected cells were incubated until a similar cytopathic effect (CPE) in the non-treated (NT) or tomatidine-treated wells was observed and then further passaged on a fresh monolayer of Huh7 cells. For each treatment group, four biological replicates were performed (A-D). In the second approach, the first virus passage was performed at MOI 1 on Huh7 cells treated with 10 μM tomatidine, EtOH or plain medium. After 24 h, the virus-containing supernatant was removed and used to infect a fresh monolayer of Huh7 cells. For virus passages two to seven, 45 μL (corresponds to a 1:10 dilution) of supernatant was used to re-infect the NT and EtOH control and 450 μL of supernatant was used to re-infect the tomatidine samples. A high volume of virus inoculum was added for tomatidine-treated samples to correct for the antiviral effect. During each passage, the virus inoculum was removed at 2 hpi, cells were washed three times and medium containing the corresponding compound was added. The virus was passaged for a total of 7 times and three biological replicates were performed for each treatment group (A-C). The CHIKV passage (p) 15 and CHIKV p7 viruses produced in the respective studies were titrated by plaque assay. Each produced virus-stock was then used to infect Huh7 cells at MOI 1 and treated with 10 μM tomatidine or the equivalent volume of EtOH. Supernatants were collected at 9 hpi and plaque assay was used to determine with infectious virus titer.

### ATPLite

The cytotoxic effect of ivermectin on Huh7 cells was evaluated with use of the ATPLite Luminescence Detection assay system. Huh7 cells were seeded in a white polystyrene 96-well plate at a density of 0.8*10^3^ cells per well. At 24 h post-seeding, cells were treated with increasing concentrations of ivermectin ranging from 2 to 40 μM and incubated for 24 h. Subsequently, ATPLite assay was performed and luminescence was detected with a microplate reader (Biotek, Sinergy, HT, Vermont, USA) [17]. Cytotoxicity was expressed as indicated below and values were plotted as percentage compared to a NT control.


$$\%\;Cytotoxicity=\frac{\left(Abs\;sample)-(Abs\;blank\right)}{\left(Abs\;negative\;control)-(Abs\;blank\right)}*100$$

### Statistical analysis

The ivermectin concentration causing 50% cytotoxicity is defined as CC50 and was determined by a dose-response curve fitted by non-linear regression analysis (sigmoidal model). Data was analyzed using GraphPad Prism (La Jolla, CA, USA) and presented as ±SEM. The tests used for statistical analysis are described in each figure legend. A p value ≤ 0.05 was considered significant (*p≤ 0.05, **p≤ 0,01, ***p≤0.001, ****p≤0.0001).

## Results

### Tomatidine induces a limited number of proteomic changes in Huh7 cells

**Fig 1:**
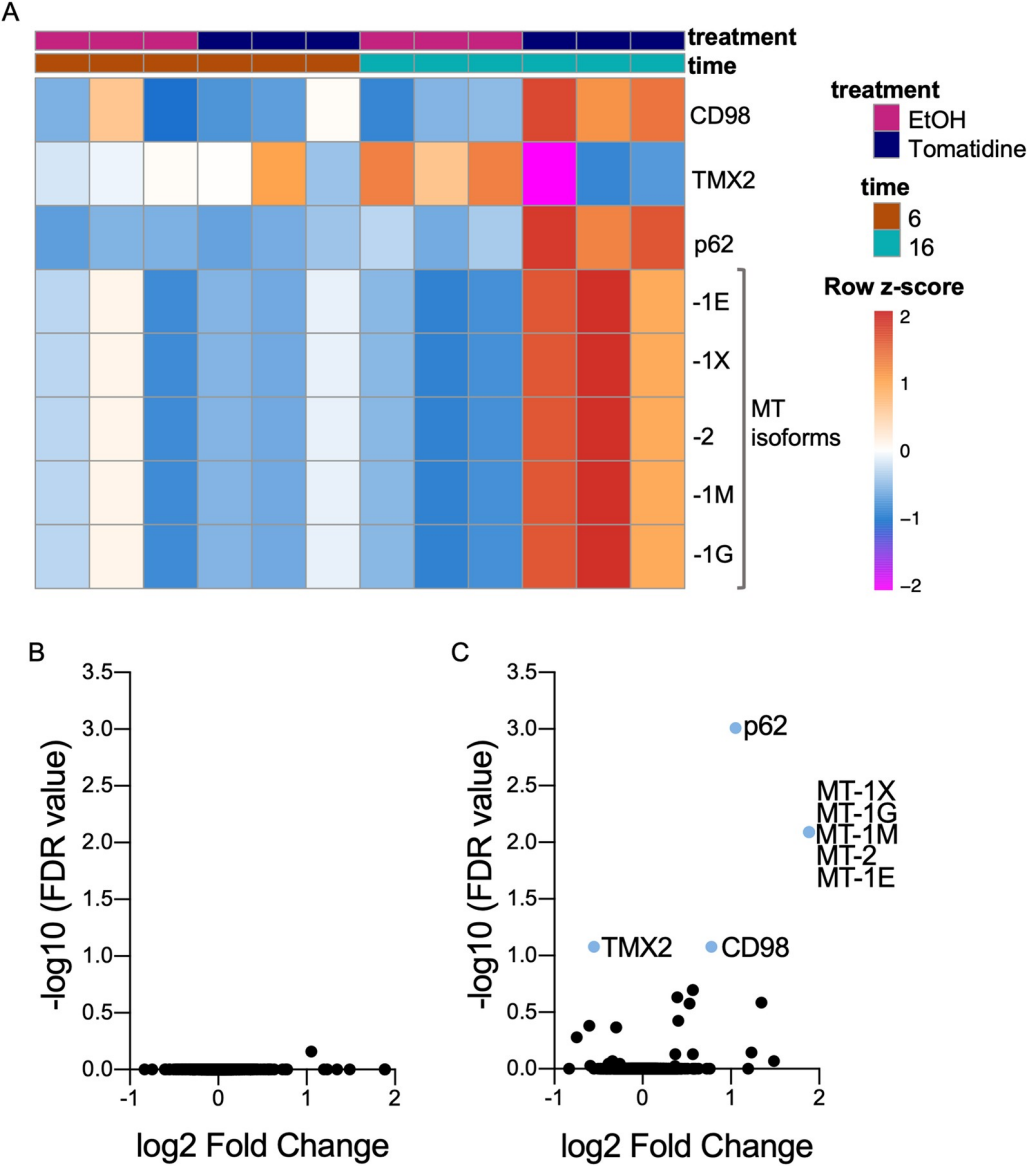
Tomatidine induces proteomic changes in Huh7 cells. Huh7 cells were treated with 10 μM tomatidine or the equivalent volume of EtOH and collected after an incubation time of 6 and 16 h. Proteins were isolated and samples were prepared for mass spectrometry analysis. (A) Supervised heat-map of all differentially expressed proteins in the presence of tomatidine compared to the EtOH-treated control. (B-C) Differential protein expression in the presence of tomatidine compared to EtOH-treated cells at 6 hpi (B) and 16 hpi (C) presented in a volcano plot. Proteins were regarded differentially expressed with a false discovery rate (FDR) lower than 0.1. Data from three independent experiments are presented.

Tomatidine may exert its antiviral activity towards CHIKV by modulating one or multiple cellular factors required for CHIKV replication. To investigate this hypothesis, we first evaluated the effect of tomatidine on the host proteome by mass spectrometry. Briefly, human hepatic Huh7 cells were treated with the highest non-toxic yet antiviral dose of tomatidine (10 μM) or the equivalent volume of EtOH as solvent control [17]. Human hepatic cells were chosen as CHIKV is known to infect hepatocytes during natural infection and we previously showed potent antiviral activity of tomatidine in this cell line [17]. At 6 or 16 h post-treatment, the cellular proteins were isolated and prepared for mass spectrometry. These time-points were chosen to measure early and late proteomic changes upon tomatidine treatment in relation to the CHIKV replication cycle. In Huh7 cells, one CHIKV replication cycle lasts approximately 8 h. Our previous study showed a potent antiviral effect up to roughly three replication cycles [17]. Initial review of the data revealed that 1215 proteins were identified during the mass spectrometry analysis. Subsequent analysis revealed that the treatment groups segregated at 16 hpi but not 6 hpi (S1A and S1B Fig). In line with this, no significant proteomic changes were observed in tomatidine-treated cells at 6 h when compared to the EtOH control (Fig 1A and 1B), Table 1). At 16 h of tomatidine treatment, four cellular proteins were differentially expressed compared to the EtOH control (Fig 1A and 1C). Of these, CD98, p62 and metallothionein (MT) were significantly upregulated, whereas thioredoxin-related transmembrane protein 2 (TMX2) was significantly downregulated (Fig 1, Table 1). The strongest effect was observed for MT (3.68-fold), followed by p62 (2.07-fold), CD98 (1.72-fold), and lastly TMX2 (0.68-fold).

**Table 1:** Fold changes in protein expression.

Protein hit	6 h Toma vs EtOH	16 h Toma vs EtOH
CD98	0.95	1.72*
TMX-2	1.04	0.68*
p62	1.02	2.07*
MT	0.93	3.68*

*FDR <0.1 # Orange: upregulation; blue: downregulation

**Fig 2:**
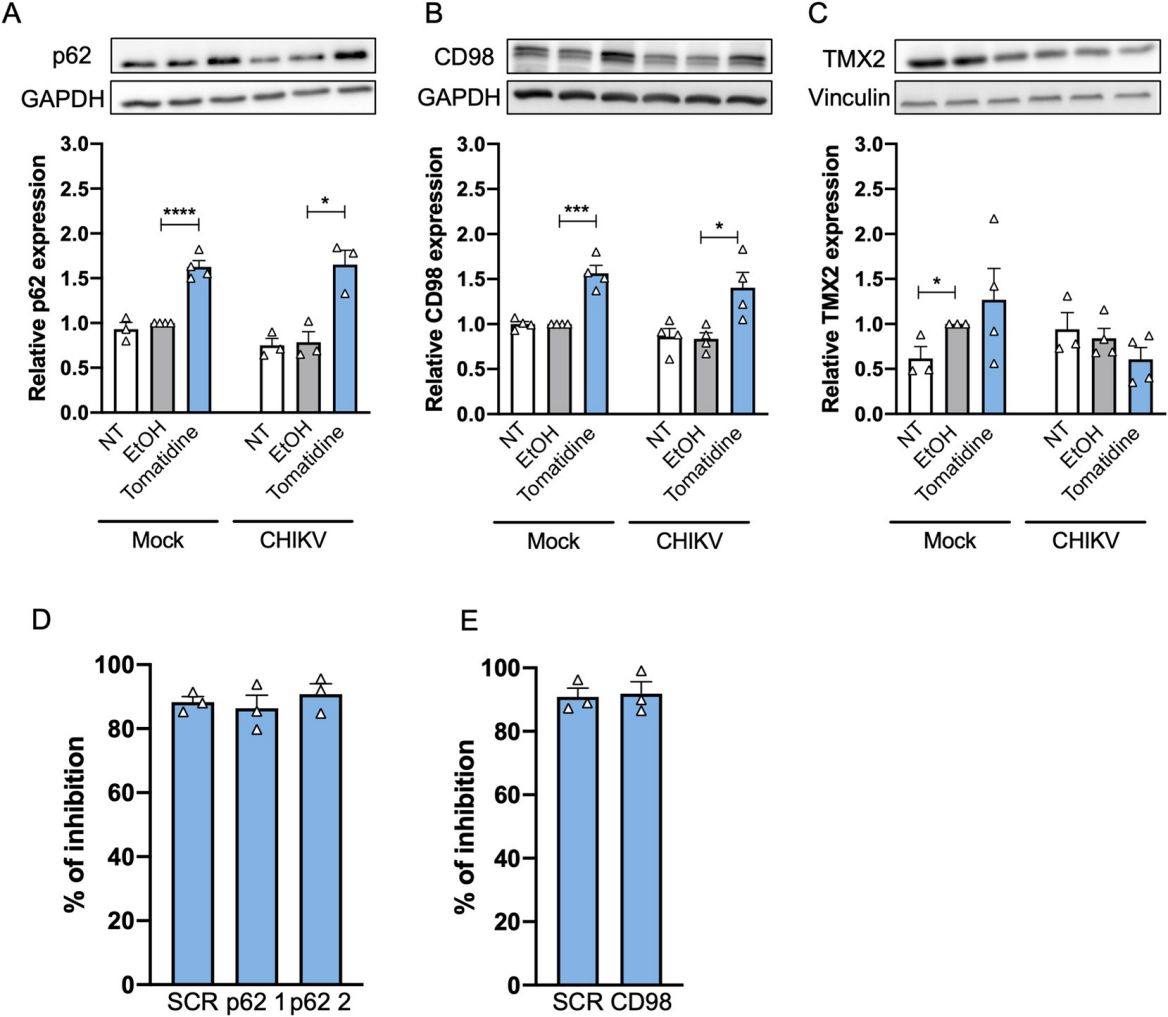
Tomatidine induces the expression of p62 and CD98. Huh7 cells were infected with CHIKV-LR at MOI 10 and simultaneously treated with 10 μM tomatidine or EtOH or left non-treated. Infected cells and supernatants were collected at 9 hpi. (A-C) Expression of (A) p62, (B) CD98 or (C) TMX2 was measured via western blot analysis. Representative blots are presented in the upper panel. Vinculin or GAPDH were used as a loading control. The lower panel shows the quantification of normalized protein expression relative to the mock-infected EtOH-treated control. (D-E) Huh7 cells were reverse-transfected with 10 nM of siRNA targeting (D) p62, (E) CD98 or a scramble control (SCR). At 48 h post-transfection, cells were infected with CHIKV-LR at MOI 10 and treated with 10 μM tomatidine, the equivalent volume of EtOH or left non-treated (NT). Supernatants were collected at 9 hpi and infectious particle production was determined via plaque assay. Fig represents the % of inhibition compared to the EtOH control. Data are presented as mean ± SEM from at least three independent experiments. The statistical significance was determined using an unpaired t-test.

To validate the protein hits and at the same time assess the effect of CHIKV on the protein expression levels, we next performed a western blot analysis in tomatidine-treated mock-infected and CHIKV-infected Huh7 cells. Hence, Huh7 cells were mock-infected or CHIKV-infected at MOI 10 and treated with 10 μM tomatidine, EtOH or left untreated. We decided to analyze the protein expression at 9 hpi as at this time point a clear antiviral effect of tomatidine is observed. Unfortunately, we were not able to validate expression of MT due to the lack of efficient antibodies. The results were normalized to the corresponding loading control (GAPDH or vinculin) and expressed as relative protein expression compared to the mock-infected, EtOH-treated control (Fig 2A and 2C). For p62 and CD98, no difference in protein expression was observed between the NT and EtOH-treated samples, demonstrating that the solvent does not induce changes in the expression of these proteins (Fig 2A and 2B). Tomatidine treatment increased p62 expression by 1.7-fold in mock-infected cells and 1.8-fold in CHIKV-infected samples when compared to the solvent control. For CD98, a 1.6-fold increase in the mock-infected samples and a 1.4-fold increase in the CHIKV-infected samples was observed upon tomatidine treatment (Fig 2B). For TMX2, a significant difference between the NT and EtOH-treated samples was seen during mock-infection. However, the presence of tomatidine did not induce any significant change in the protein expression of TMX2 (Fig 2C). Altogether, we validated p62 and CD98, which are both upregulated upon tomatidine treatment irrespective of whether the cells are infected with CHIKV or not.

### CD98 and p62 are not involved in the anti-Chikungunya virus activity of tomatidine

We next evaluated whether the upregulation of CD98 and p62 plays a role in the antiviral mechanism of tomatidine. Hence, we performed a siRNA knockdown of CD98 and p62 in Huh7 cells using reverse transfection. A non-coding scramble siRNA (SCR) was used as control. At 48 h post-transfection, Huh7 cells were infected with CHIKV at MOI 10 and treated with 10 μM tomatidine, EtOH or left untreated. At 9 hpi, expression of CD98 and p62 and the production of progeny virus was determined. The expression of CD98 and p62 was normalized to the loading control and displayed as relative expression compared the mock-infected NT SCR control. Upon transfection with the siRNA, the average CD98 protein level was significantly reduced by 3.2-fold when compared to the SCR control (S2A Fig). For cells transfected with p62 siRNAs #1 and #4, the average p62 level was reduced by 3.3-fold and 3.5-fold compared to the SCR control, respectively (S2B Fig). All original western blot scans of S2 Fig are depicted in S3 Fig. Despite the efficient knockdown of CD98 and p62, tomatidine was still found highly antiviral towards CHIKV (Fig 2D and 2E). For cells with knock-down of CD98 and p62 expression, the inhibition of infectious particle production was 91.68% and 86.37% (siRNA #1) or 90.82% (siRNA #4) respectively. This was similar to the inhibitory effect observed in cells treated with the corresponding SCR controls (Fig 2D and 2E). Thus, p62 and CD98 are not involved in the antiviral potency of tomatidine towards CHIKV in Huh7 cells.

### Tomatidine does not affect Chikungunya virus cell entry

**Fig 3:**
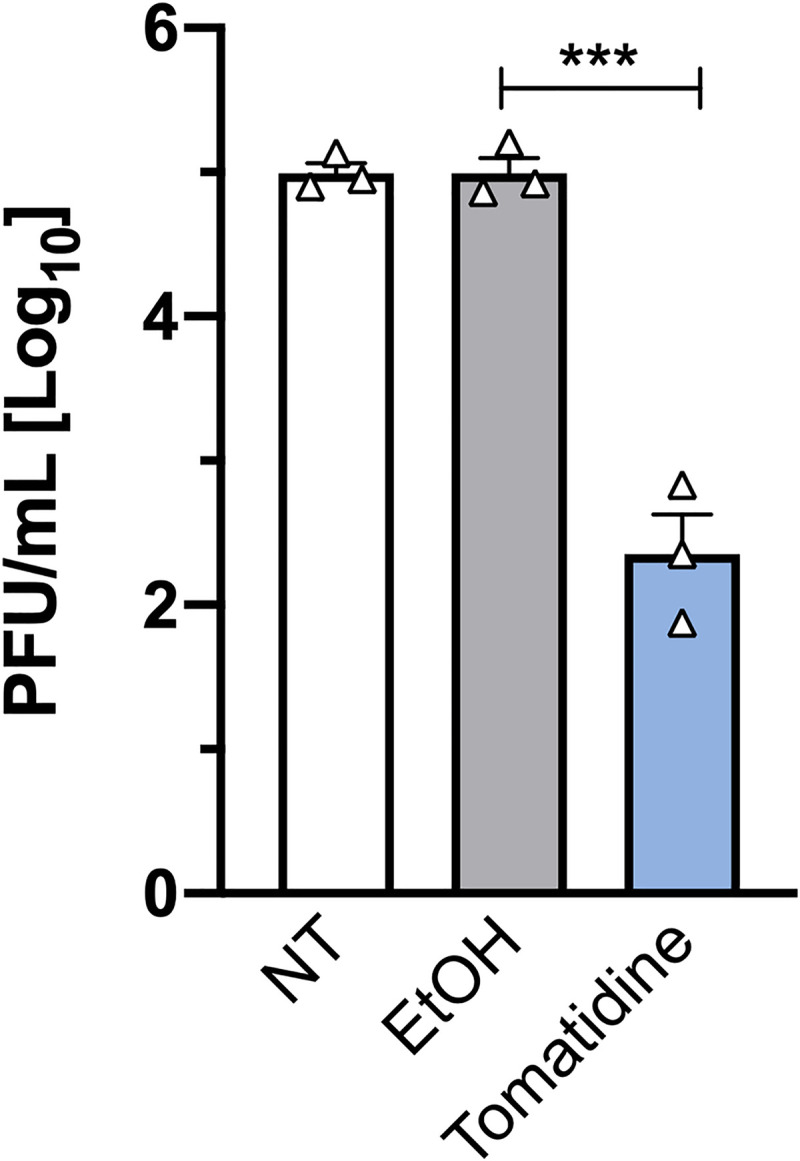
Tomatidine acts at a post-entry step of the virus replication cycle. Huh7 cells were transfected with CHIKV *in vitro* transcribed RNA via electroporation. Transfected cells were incubated in the presence of 10 μM tomatidine, the equivalent volume of EtOH or left non-treated (NT). Supernatants were collected at 16 h post-transfection and infectious virus particle production was determined via plaque assay. Data are presented as mean ± SEM from three independent experiments. The statistical significance was determined using an unpaired t-test.

Next, we focused on elucidating the steps of the virus replication cycle that are inhibited by tomatidine. The time-of-addition experiment presented in our previous study suggests that tomatidine acts after virus attachment and entry into the host cell as the most pronounced antiviral effect was seen when the compound was added at post-entry conditions [17]. To validate this finding, we here performed a virus cell entry-bypass assay. Hereto, *in vitro* transcribed RNA was directly electroporated into cells which were subsequently treated with 10 μM tomatidine, the equivalent volume of EtOH or left untreated. At 16 h post-transfection, the infectious virus titer was determined via plaque assay. The 16 h time-point was chosen to provide sufficient recovery time for the cells after the electroporation procedure. Previous data showed a significant antiviral effect of tomatidine at 16 hpi at normal infection conditions [17]. Upon electroporation of *in vitro* transcribed viral RNA, non-treated (NT) cells produced 5.0±0.1 Log10 PFU/mL (Fig 3). An identical titer (5.0±0.1 Log10 PFU/mL) was observed for the EtOH-treated cells. In the presence of tomatidine, however, the virus titer was significantly reduced to 2.4±0.3 Log10 PFU/mL, which corresponds to a 2.6-log reduction (Fig 3). Thus, tomatidine still exhibits potent antiviral activity when virus cell entry is bypassed which confirms that tomatidine targets a step after the virus cell entry process.

### Tomatidine reduces the copy numbers of Chikungunya virus RNA

**Fig 4:**
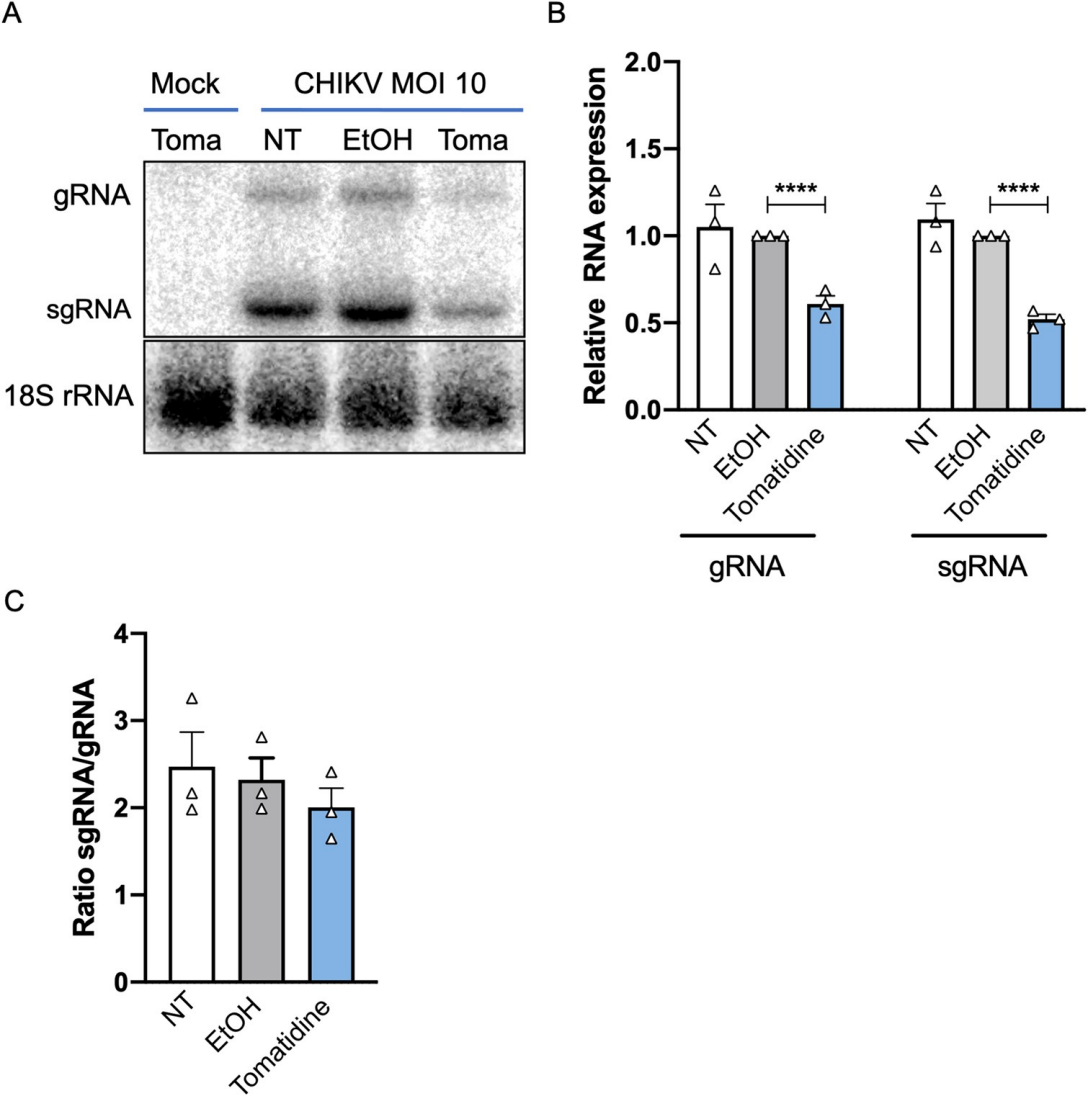
Tomatidine reduces the copy numbers of the gRNA and sgRNA of CHIKV. Huh7 cells were infected with CHIKV-LR at MOI 10 and simultaneously treated with 10 μM tomatidine, the equivalent volume of EtOH or left untreated. At 6 hpi total RNA was isolated from infected cells. In gel hybridization was performed to detect the gRNA and sgRNA of CHIKV. (A) Representative scan of the gel after labelling with a ^32^P-labeled CHIKV probe. (B) Quantification of the bands detected via in gel hybridization. Samples were normalized to 18S rRNA and expressed as relative expression to the EtOH-treated control. (C) The sgRNA/gRNA ratio based on the results presented in A/B. Data are presented as mean ± SEM from three independent experiments. The statistical significance was determined using an unpaired t-test.

Upon virus cell entry, the genomic RNA is translated and genome replication is initiated. We first used a *trans*-replicase system to investigate RNA replication in an isolated manner. Hereto, two plasmids were co-transfected into Huh7 cells. The first plasmid encodes a replication competent RNA template containing the 5’ and 3’ untranslated regions of the CHIKV genome and a sequence encoding fluorescent tomato marker placed under the control of CHIKV subgenomic promoter. The second plasmid encodes the CHIKV replicase, which is capable of replicating the template RNA and allows for the transcription of the mRNA of the fluorescent marker protein. Thus, co-transfection of both plasmids facilitates replication and the expression of the fluorescent tomato marker is indicative of RNA replication. As a control, a non-functional replicase plasmid (GAA) was used. Moreover, ivermectin, a previously reported antiviral compound described to inhibit CHIKV replication, was included as a positive control [35]. At 1 h post-transfection, 10 μM tomatidine, the equivalent volume of EtOH or 7 μM ivermectin were added to the cells. The selected ivermectin concentration was shown to be non-toxic in Huh7 cells via an ATPLite assay (S4A Fig). At 24 h post-transfection, the expression of the fluorescent tomato marker was analyzed via flow cytometry. As expected, co-transfection of the template-expressing plasmid with the non-functional replicase plasmid (GAA) did not yield any fluorescent signal (S4B Fig). In contrast, co-transfection with the functional replicase plasmid led to 7.55±0.75% and 8.18±0.45% fluorescent tomato-expressing cells, in NT and EtOH-treated samples, respectively (S4B Fig). Treatment with the positive control ivermectin reduced the percentage of tomato-expressing cells to 2.19±0.5%, which corresponds to a reduction of 73% compared to the EtOH control. Tomatidine treatment reduced the percentage of marker protein expressing cells to 4.4±0.69%, which represents a reduction by 46% compared to the EtOH control. Hence, tomatidine inhibits the RNA replication in a CHIKV *trans*-replicase system, albeit to a lesser extent than ivermectin. In this assay, we also determined the mean fluorescent intensity (MFI) of the tomato marker within the marker-positive cell population and expressed the data as MFI relative to the EtOH control. Ivermectin treatment significantly reduced the MFI to 0.4±0.06 (S4C Fig). For tomatidine, however, no effect on the MFI was observed (1.0±0.06), indicating that tomatidine reduces the initiation of replication but once replication is established within the cell, tomatidine can no longer interfere with the process. Hereafter, we aimed to investigate the effect of tomatidine on RNA replication under CHIKV infection conditions. First, we visualized the positive-sense genomic RNA (gRNA) and subgenomic RNA (sgRNA) via in-gel hybridization. The sgRNA is produced later during the infection process and contains the 3’ ORF encoding the structural proteins needed to produce progeny virions [2]. Huh7 cells were infected with CHIKV at MOI 10 and treated with 10 μM tomatidine, the equivalent volume of EtOH or left non-treated. At 6 hpi, the total RNA was isolated from infected cells and in gel-hybridization was performed. As loading control, the 18S ribosomal RNA (rRNA) was used (Fig 4A). Results were normalized to the loading control and expressed as relative RNA level compared to the EtOH control (Fig 4B). Upon CHIKV infection, both gRNA and sgRNA was detected and no difference was seen in RNA levels between the NT and EtOH samples (Fig 4A and 4B). Importantly, a clear reduction in gRNA (1.7-fold, corresponding to 40%) and sgRNA (2-fold, corresponds to 50%) band intensity was observed for the tomatidine-treated samples when compared to the EtOH control (Fig 4A and 4B). To assess whether tomatidine interferes with the specific expression of one of the two viral RNAs, we next determined the sgRNA to gRNA ratio (Fig 4C). No statistical differences were observed in the presence or absence of tomatidine, indicating that tomatidine interferes with the overall expression of gRNA and sgRNA rather than with the synthesis of gRNA or sgRNA, in particular.

**Fig 5:**
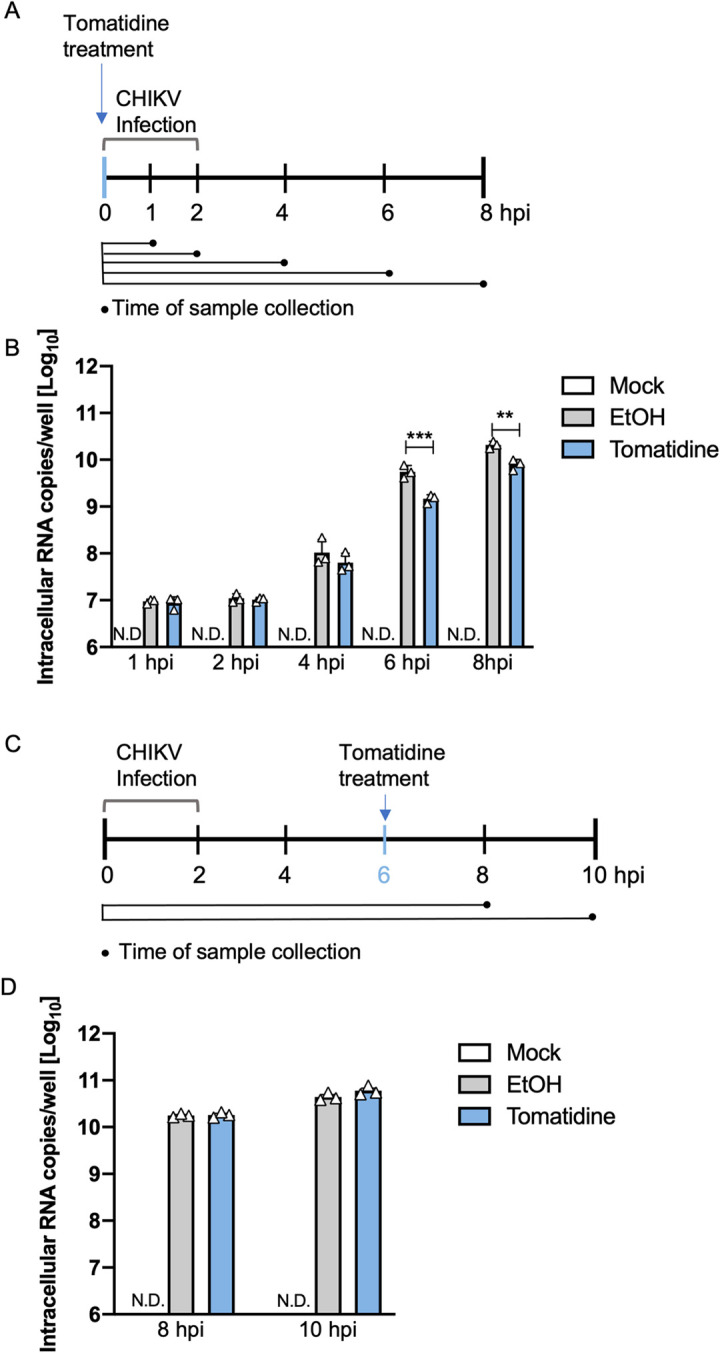
Tomatidine reduces the abundance of intracellular CHIKV RNA at 6 and 8 hpi. (A-B) Huh7 cells were infected with CHIKV-LR at MOI 10. At the time of infection, cells were treated with 10 μM tomatidine or the equivalent volume of EtOH. As a control mock-infected cells were used. Total RNA was isolated at 1, 2, 4, 6 and 8 hpi and Q-RT-PCR was performed to determine the intracellular CHIKV RNA copies per well. (A) Outline of the experiment. (B) Intracellular RNA copies per well presented on a Log10 scale. (C-D) Huh7 cells were infected with CHIKV-LR at MOI 10 and treated with tomatidine or the equivalent volume of EtOH at 6 hpi. Again, mock-infected cells were used as control. RNA from infected cells were isolated at 8 and 10 hpi and CHIKV RNA copy numbers per well were determined via Q-RT-PCR. (C) Experimental outline. (D) Intracellular RNA copies per well on a Log10 scale. N.D. refers to non-detectable, which means that the samples were below the detection limit (250 RNA copies). Data are presented as mean ± SEM from three independent experiments. Significance was determined using a 2way ANOVA with Bonferroni’s multiple comparisons test.

To gain a better understanding of the dynamics and timing of the inhibitory effect of tomatidine, we next infected Huh7 cells in the presence or absence of tomatidine with CHIKV at MOI 10 and determined the number of intracellular viral RNA copies at 1, 2, 4, 6 and 8 hpi (Fig 5A). For the mock-infected samples, the detected viral RNA copies fell below the threshold of detection (N.D.) (Fig 5B). For the CHIKV-infected samples, the amount of viral RNA increased over time and reached 10.3±0.1 Log10 RNA copies at 8 hpi (Fig 5B). At 1 and 2 hpi, the RNA load remained constant (~7 Log10 copies) for all experimental conditions, which suggests that these numbers reflect RNA released from incoming virus particles (Fig 5B). At 4 hpi, the RNA load increased to 8.0±0.2 Log10 copies in the EtOH sample and 7.8±0.1 Log10 copies in the tomatidine-treated samples (Fig 5B). At 6 hpi and 8 hpi, tomatidine treatment significantly reduced the RNA load compared to the EtOH control. At 6 hpi the RNA load was reduced from 9.7±0.1 to 9.2±0.1 Log10 copies (corresponds to a 74% reduction) and at 8 hpi the intracellular RNA load was reduced from 10.3±0.0 to 9.9±0.1 Log10 copies (corresponds to a 62% reduction) (Fig 5B). In conclusion, tomatidine treatment was found to significantly reduce the intracellular levels of CHIKV RNA genome at 6 and 8 hpi.

### Tomatidine does not interfere with ongoing viral RNA replication

In Fig 5B, tomatidine was added at the time of infection thus prior to the onset of viral RNA replication. To assess whether tomatine can interfere with ongoing viral RNA replication, we next added tomatidine to cells at 6 hpi and evaluated the viral RNA copy numbers at 8 and 10 hpi (Fig 5C). We decided to add tomatidine at 6 hpi as the results in Fig 5B show that active replication is still ongoing at this time point and because previous time of addition experiments [17] revealed that the presence of tomatidine between 6 and 9 hpi significantly reduced virus progeny production. As before, for the mock-infected samples no viral RNA was detectable (N.D.) (Fig 5D). In the absence of tomatidine, an intracellular viral RNA copy number of 10.3±0.0 (8 hpi) and 10.6±0.0 (10 hpi) Log10 was obtained. Comparable results were obtained in the presence of tomatidine (Fig 5D). Hence, when added during active RNA replication, tomatidine is not able to interfere with viral RNA replication, indicating that tomatidine cannot interfere with ongoing RNA replication.

### Tomatidine reduces the expression of Chikungunya virus proteins

**Fig 6:**
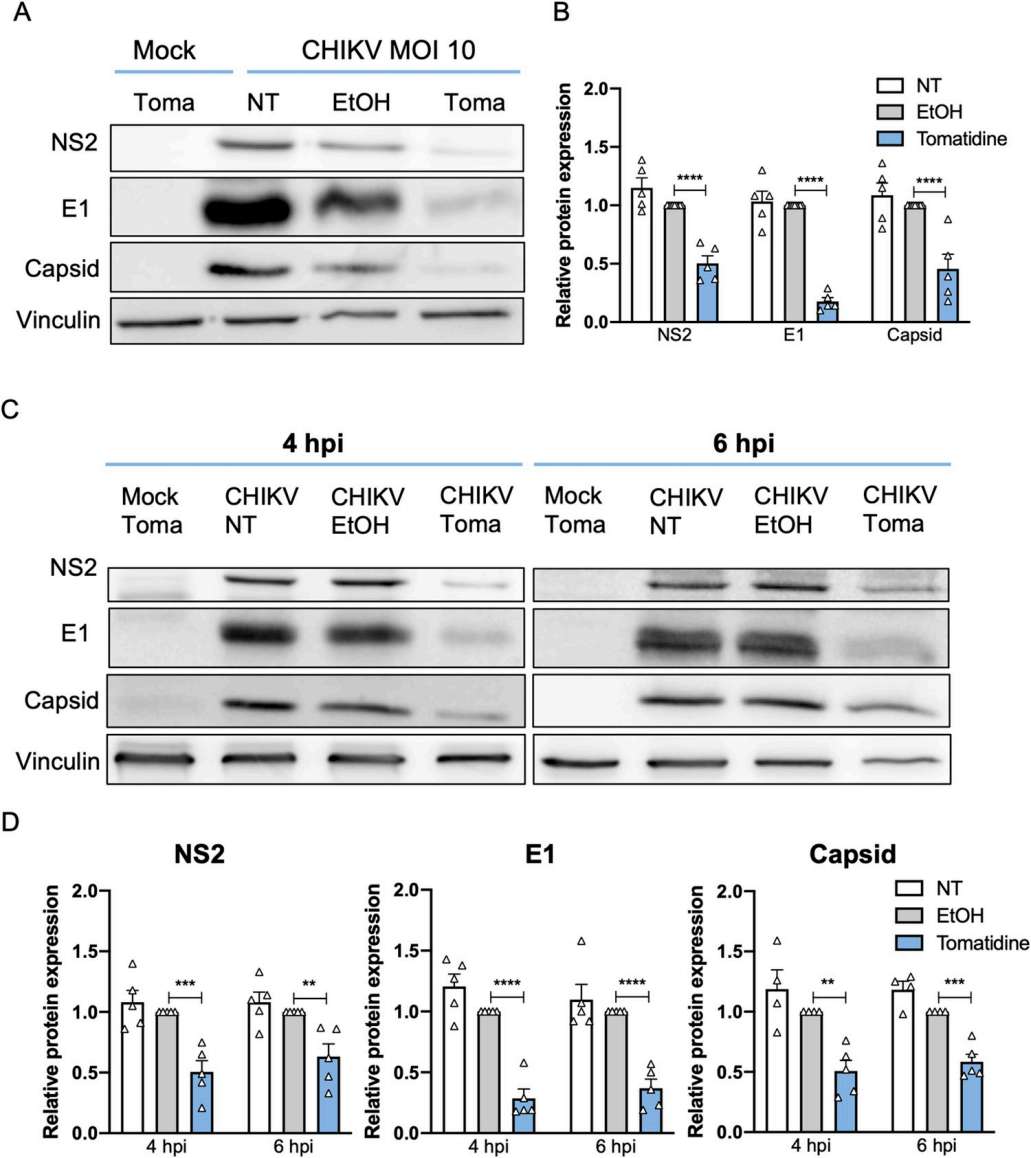
Tomatidine reduces the expression of CHIKV proteins. Huh7 cells were infected with CHIKV-LR at MOI 10 and treated with 10 μM tomatidine, the equivalent volume of EtOH at different time points or left untreated (NT). Cells were collected at 9 hpi. Western blot analysis was performed to detect the viral nsP2, E1 and capsid (C), as well as vinculin as a loading control. Band densities of viral proteins were normalized to vinculin and expressed as relative protein expression compared to the CHIKV-infected EtOH control. (A-B) Cells were treated with tomatidine or EtOH simultaneous to infection. (A) Representative western blot. (B) Quantification of the normalized protein expression relative to the CHIKV-infected EtOH control. Significance was determined using one sample t and Wilcoxon test. (C-D) Cells were treated with tomatidine or EtOH at 4 or 6 hpi. (C) Representative western blot and (D) quantification of the normalized protein expression relative to the CHIKV-infected EtOH control. Significance was determined using a 2way ANOVA with Bonferroni’s multiple comparisons test. Data are presented as mean ± SEM from five independent experiments.

Next, to test whether the reduction in CHIKV particle production upon tomatidine treatment is attributed to an effect on viral protein expression, we evaluated the expression levels of the CHIKV nsP2, E1 and capsid (C) proteins at 9 hpi upon tomatidine treatment at different time-points (0, 4 and 6 hpi) using western blot analysis. In these experiments, the protein expression levels were determined at 9 hpi to allow for sufficient protein production for detection. Vinculin was used as a loading control (Fig 6A and 6C). The observed nsP2, E1 and C band densities on the western blot were normalized to that of vinculin and expressed as fold-change to the EtOH control (Fig 6B and 6D). Comparable viral protein expression was observed between the NT and EtOH-treated samples for all detected proteins at all time points, indicating that EtOH does not affect the viral protein expression levels (Fig 6A and 6D). When tomatidine was added simultaneous to infection, the nsP2, E1 and C expression was reduced to 0.5±0.1 (50%), 0.18±0.1 (18%) and 0.46±0.1 (46%) respectively, when compared to the EtOH control (Fig 6A and 6B). A comparable pattern was seen when tomatidine was added at 4 hpi (Fig 6C and 6D). Here, tomatidine reduced the protein expression to 0.5±0.1 (50%) for nsP2, 0.3±0.1 (30%) for E1 and 0.5±0.2 (50%) for C. Importantly, the addition of tomatidine at 6 hpi still had an effect on viral protein expression (Fig 6C and 6D), Under these conditions, nsP2 was reduced to 0.6±0.2 (60%), E1 was reduced to 0.4± 0.2 (40%) and C was reduced to 0.6±0.1 (60%) compared to the corresponding EtOH control. The results demonstrate that tomatidine significantly reduces the expression of the CHIKV proteins during infection when added at the time of infection but also when added during active CHIKV replication (i.e. at 4 and 6 hpi).

### Chikungunya virus does not readily develop resistance towards tomatidine

**Fig 7:**
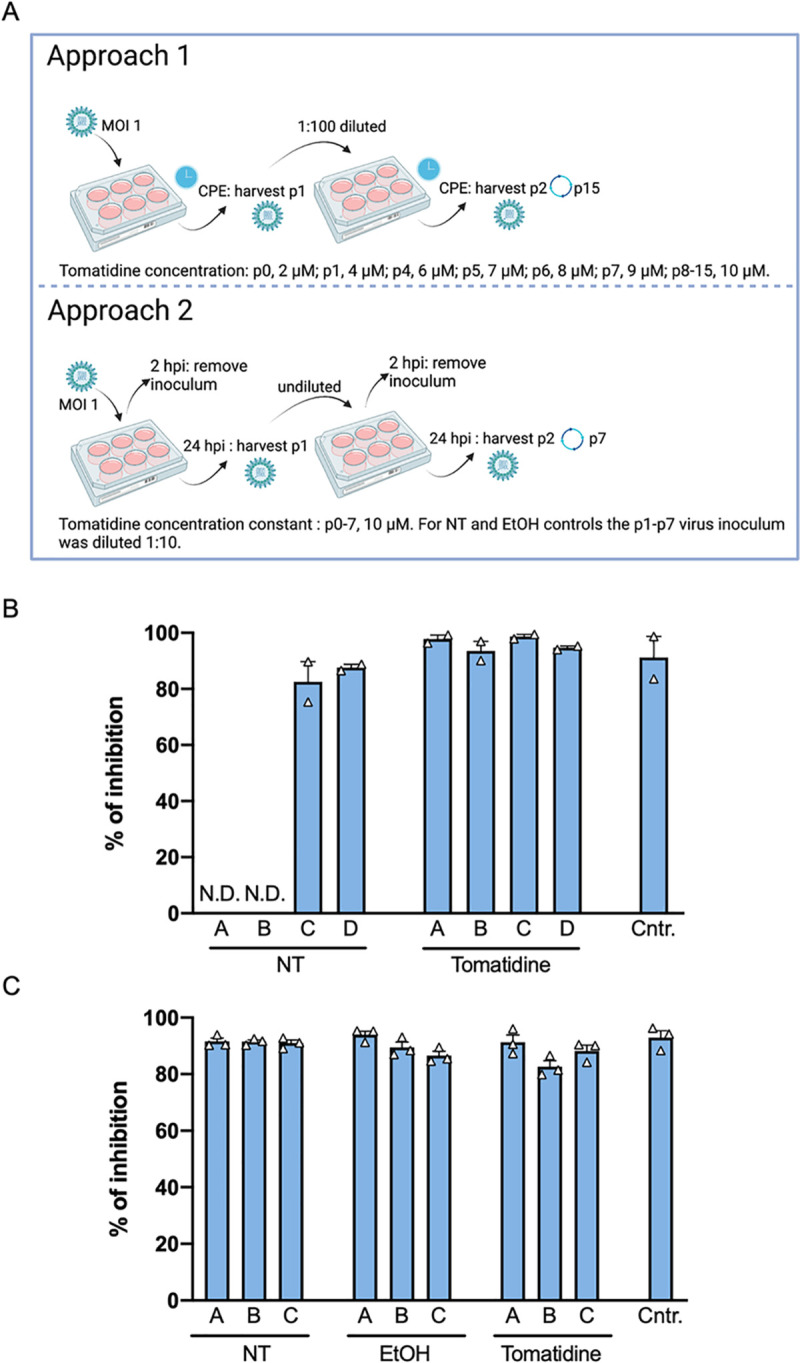
CHIKV does not readily develop resistance to tomatidine. (A) Schematic overview of the different experimental setups. Fig was created by the authors in Biorender.com. (B-C) Huh7 cells were infected with the (B) passage 15 or (C) passage 7 CHIKV samples in the presence of EtOH, tomatidine or in the absence of any compound at MOI 1. At the time of infection, cells were treated with 10 μM tomatidine or the equivalent volume of EtOH. The parental CHIKV strain was used as an internal control. Supernatants were collected at 9 hpi and infectious particle production was measured via plaque assay. Data is presented as percentage of inhibition of infectious particle production compared to the EtOH control. (A) A, B, C and D refer to the biological replicates. Data is presented as mean ± SEM from two independent experiments. (B) A, B and C represent the biological replicates. Data are presented as mean ± SEM from three independent experiments. The statistical significance was determined using an unpaired t-test.

Resistance to antiviral compounds is often described. This is particularly true for RNA viruses, since the RNA-dependent RNA polymerase lacks proof-reading activity and has a mutation rate of 10^−6^–10^−4^ substitutions per nucleotide per cell infection [36]. To test resistance development of CHIKV towards tomatidine, we applied two frequently used sequential passaging approaches presented in Fig 7A. In the first approach, CHIKV was passaged in the presence of increasing tomatidine concentrations on Huh7 cells for 15 passages. More specifically, with each passage (p), a higher tomatidine concentration was added starting from 2 to 4, 6, 7, 8, 9 and lastly 10 μM (Fig 7A). Passage 8 till p15 was performed in presence of 10 μM tomatidine. As a control, CHIKV was passaged 15 times in the absence of tomatidine (NT). In this set-up, the virus inoculum was not removed. Cell culture supernatants were collected once the cytopathic effect (CPE) between the NT and tomatidine-treated samples was similar. The obtained virus stocks were titrated and then used in an infection experiment (MOI 1) in Huh7 cells treated with tomatidine or the EtOH control. For the p15 CHIKV of NT stocks, two of the replicates (A and B) were lost in the passaging process (N.D., Fig 7A). The remaining p15 NT stocks, the p15 tomatidine-treated virus stocks and the parental CHIKV control were highly sensitive to tomatidine treatment (Figs 7B and S5A). For all samples, the inhibitory effect of tomatidine varied between 82% and 99% (Fig 7B). Thus, in this approach, CHIKV did not develop resistance towards tomatidine.

In the second approach, we sequentially passaged the virus in the presence of 10 μM tomatidine, the equivalent volume of EtOH or under NT conditions on Huh7 cells for a total of 7 passages. Here, the virus inoculum was removed after 2 h and fresh compound-containing medium was added to the cells (Fig 7A). The supernatants were collected every 24 h. After seven passages, the virus stocks were titrated using plaque assay and used in an infection experiment at MOI 1. Irrespective of the treatment condition, the p7 virus stocks remained highly sensitive towards tomatidine treatment (Figs 7C and S5B) as the inhibitory effect of tomatidine varied between 82% and 94% (Fig 7C). Hence, the sequential passaging of CHIKV in the presence of 10 μM tomatidine did not lead to the development of resistance. Altogether, we did not observe resistance development of CHIKV towards tomatidine highlighting the potential of tomatidine as an antiviral treatment option for CHIKV infection.

## Discussion

**Fig 8:**
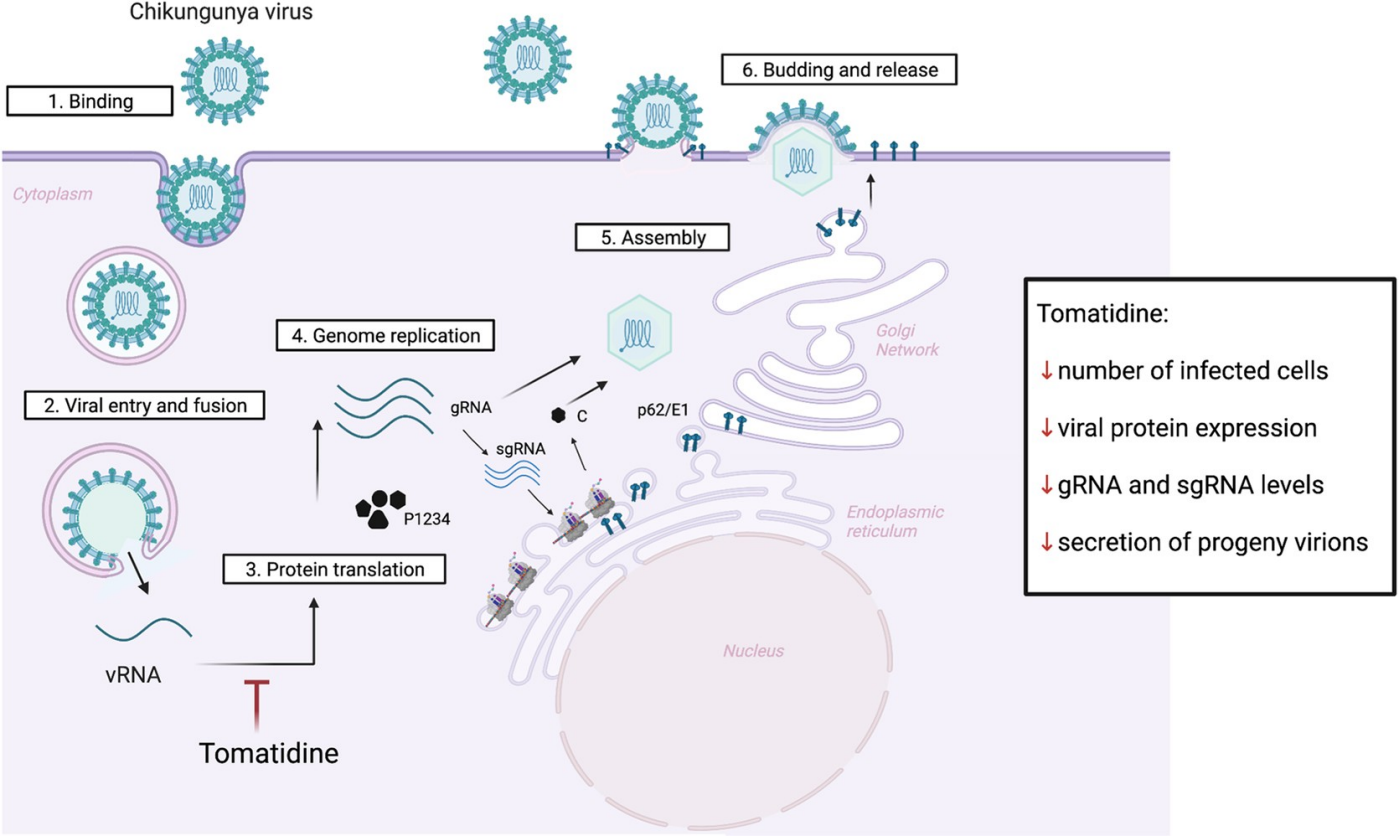
Effect of tomatidine on the CHIKV replication cycle. After binding of the virus to cell surface receptors (1), the virus is internalized and fuses from within early endosomes to release the nucleocapsid (2). The viral RNA is translated to produce P1234 (3), a polyprotein which upon processing facilitates genome replication (4). Thereafter, replication of a subgenomic RNA occurs which is translated into the structural proteins C, p62-E1. The C together with progeny genomic RNA form a nucleocapsid (5). The p62-E1 structural proteins are processed during transit via the secretory pathway and are finally expressed at the cell surface where virus assembly (5) and budding (6) occurs. Based on the obtained results (box) we hypothesize that tomatidine actively interferes with protein translation which reduces viral genome replication thereby limiting the chance to productively infect a cell. The effect of tomatidine on protein translation culminates in a reduced secretion of progeny virions. Fig was created by the authors in Biorender.com.

In this study we investigated the mechanism by which tomatidine interferes with CHIKV infectivity. Initial mass spectrometry analysis in Huh7 cells revealed that tomatidine significantly controls the expression of four cellular proteins. CD98 and p62 were validated yet subsequent siRNA knockdown experiments revealed that these proteins are not responsible for the observed antiviral effect of tomatidine towards CHIKV. In parallel, the effect of tomatidine on the CHIKV replication cycle was studied and the results are summarized in Fig 8. Potent antiviral activity was seen when *in vitro* transcribed CHIKV RNA was transfected into Huh7 cells treated with tomatidine. Furthermore, tomatidine treatment was found to significantly inhibit the number of CHIKV intracellular RNA copies at 6 and 8 hpi. The levels of gRNA and sgRNA were equally reduced. Also, a clear inhibition of viral nsP2, E1 and the C protein expression was observed at 9 hpi when tomatidine was added simultaneous to infection but also when added 4 and 6 hpi. Intriguingly, however, no effect on the RNA copy number was seen when tomatidine was added during active viral replication, a finding that was supported by data obtained using the CHIKV *trans-*replicase system. Lastly, sequential passaging of CHIKV on Huh7 cells in the presence of tomatidine did not induce viral resistance towards tomatidine.

Previous time-of-addition experiments [17] and the *in vitro* RNA transfection experiments presented in this study exclude an inhibiting role of tomatidine during virus cell entry [17]. Indeed, the strongest antiviral effect is seen when the compound is added at post-infection conditions [17]. Furthermore, the addition of tomatidine to cells was shown to reduce the ability of the virus to productively infect cells [17]. Subsequent experiments revealed that tomatidine treatment reduces the intracellular viral RNA levels yet tomatidine cannot interfere with ongoing RNA replication. Lastly, tomatidine was shown to reduce viral protein expression levels when added both early and late in infection. This suggests that tomatidine interferes with RNA translation or facilitates enhanced degradation of viral proteins. Taken together, and based on the above findings, we hypothesize that tomatidine controls viral protein expression early in infection thereby hampering efficient RNA replication and thus the chance to productively infect a cell. Cells that are infected may also have reduced protein expression levels as tomatidine was still able to interfere with viral protein expression levels when added at 6 hpi. Thus, we propose that the effect of tomatidine on viral protein expression level early and late in infection culminates in reduced secretion of progeny virus particles.

Cellular proteins play crucial roles in the replicative cycle of viruses. Furthermore, the high barrier of CHIKV resistance to tomatidine also suggests that tomatidine exert its antiviral activity through the modulation of cellular proteins or other host factors. By use of mass spectrometry, we aimed to unravel whether host proteins are responsible for the observed antiviral effect of tomatidine. Notably, we only found a limited number of proteomic changes in cells treated with tomatidine. This was unexpected given the numerous pathways that have been reported to be modulated by tomatidine [20–27]. It might be possible that we missed less pronounced proteomic changes as the unlabeled mass spectrometry approach we used picks-up major changes in the host cell proteome [37]. Importantly, however, the validated individual protein hits were not responsible for the observed antiviral effect of tomatidine towards CHIKV. Whether or not simultaneous downregulation of both proteins or other as yet unknown cellular proteins may affect the antiviral activity of tomatidine towards CHIKV remains to be elucidated.

Alternatively, the inhibitory activity may not be related to protein expression but rather to the location or functionality of a protein. With regard to protein functionality, metabolomic studies may help to identify cellular process that may be inhibited or modulated by tomatidine. One example could be the host cellular lipid metabolism, which has been reported to play a key role in alpha- and flavivirus infectivity, translation, replication and morphogenesis [38,39]. During replication, the virus induces vesicle spherules consisting of host-derived cellular membranes where replication and translation of the viral RNA occurs [2]. Since it has been reported that tomatidine ameliorates atherosclerosis-associated hyperlipidemia *in vivo* it may be possible that tomatidine modulates the lipid metabolism to exert its antiviral activity [24]. Future label-based mass spectrometry analysis combined with immunofluorescence analysis are needed to detect not only the less profound changes in the protein expression but also their cellular localization. This, together with metabolomic and lipidomic studies will facilitate to unveil the importance of cellular host proteins and pathways in the antiviral activity of tomatidine.

RNA viruses are prone to mutations due to the lack of proof-reading activity and therefore can quickly develop resistance towards an antiviral treatment [40]. As an example, CHIKV resistance towards arbidol occurs by one single amino acid substitution in the viral E2 protein and therefore the virus has a low genetic barrier to resistance [41]. In this study, we used two commonly used approaches to test if tomatidine treatment induces resistance. The results show that passaging of CHIKV under selection pressure of tomatidine does not readily lead to viral resistance, which is an essential characteristic of an antiviral compound and strengthens the potential use of tomatidine as an antiviral treatment towards CHIKV infection. Treatment should most likely start early after disease onset as its major function would be to reduce the viral load thereby alleviating disease symptoms.

In conclusion, we delineated that tomatidine interferes with CHIKV infectivity by modulating the expression levels of viral proteins. The exact viral and/or host factors involved in this inhibitory effect still need to be elucidated. Future studies on host cell proteomics, lipidomics, metabolomics will help to elucidate the exact mechanism by which tomatidine controls the expression of viral proteins. The ability of tomatidine to reduce CHIKV particle production and the absence of tomatidine resistance warrants further *in vivo* investigation of its pharmacological properties and antiviral efficacy to CHIKV.

## Supporting information

S1 Fig Principal component analysis of tomatidine an EtOH-treated cells. (A) Principle component analysis at 6 hpi and (B) 16 hpi.
(TIF)

S2 Fig Knockdown efficiency of siRNAs targeting p62 and CD98. Huh7 cells were reverse-transfected with 10 nM of siRNA targeting (A) CD98 or (B) p62 or a scramble control (SCR). At 48 h post-transfection, cells were infected with CHIKV-LR at MOI 10 and treated with 10 μM tomatidine, the equivalent volume of EtOH or left non-treated. Protein was collected at 9 hpi and CD98, p62 or GAPDH as a loading control were detected via western blot. Samples were analyzed side-by-side on the same gel yet for clarity the blots were split. (A) The left panel shows the representative blots for CD98 and GAPDH. The right panel displays the band quantification normalized to GAPDH and expressed as relative protein expression compared to the mock-infected, non-treated (NT) control. (B) The left panel shows the representative blots for p62 and GAPDH. The samples were loaded on distinct gels yet the proteins bands were detected at the same time and at identical conditions. The right panel displays the band quantification normalized to GAPDH and expressed as relative protein expression compared to the mock-infected, non-treated (NT) control. For p62, two different siRNAs (siRNA #1 and #4) were used. Data are presented as mean ± SEM from three independent experiments. The statistical significance was determined using one-way ANOVA Dunnett’s multiple comparisons test.
(TIF)

S3 Fig Original western blot scans of siRNA knockdown targeting CD98 and p62. (A-C) Original western blot scans of CD98 knockdown including n = 1 (A), n = 2 (B) and n = 3 (C). (D-F) Western blot scans of p62 knockdown including n = 1 (D), n = 2 (E) and n = 3 (F).
(PDF)

S4 Fig Tomatidine inhibits CHIKV replication in a *trans*-replicase system. (A) Dose-response curve of the ATP level in Huh7 cells assessed by ATPLite assay in the presence of increasing ivermectin concentrations at 24 h treatment period. (B-C) Huh7 cells were transfected with CHIKV-LR *trans*-replicase system consisting of a template plasmid including a tomato marker and a replicase plasmid or its nonfunctional mutant (GAA). At 1 h post-transfection, cells were treated with 10 μM tomatidine, the equivalent volume of EtOH or ivermectin (7 μM or 0.8 log_10_) or left non-treated (NT). At 24 h post-transfection cells were collected, fixed and analyzed via flow cytometry. (B) Percentage of transfected cells detected via the fluorescent tomato marker and (C) MFI relative to the CHIKV-infected EtOH control. Data are presented as mean ± SEM from four independent experiments. The statistical significance was determined using an unpaired t-test.
(TIF)

S5 Fig CHIKV does not readily develop resistance to tomatidine. (A-B) Huh7 cells were infected with the (A) passage 15 or (B) passage 7 CHIKV samples in the presence of EtOH, tomatidine or in the absence of any compound at MOI 1. At the time of infection, cells were treated with 10 μM tomatidine or the equivalent volume of EtOH. The parental CHIKV strain was used as an internal control. Supernatants were collected at 9 hpi and infectious particle production was measured via plaque assay. Data is presented as Log_10_ PFU per mL. (A) A, B, C and D refer to the biological replicates. Data is presented as mean ± SEM from two independent experiments. (B) A, B and C represent the biological replicates. Data are presented as mean ± SEM from three independent experiments. The statistical significance was determined using an unpaired t-test.
(TIF)
