# Supplementary figures and images for: Tomatidine reduces Chikungunya virus progeny release by controlling viral protein expression
Source: PLoS Negl Trop Dis. 2021 Nov 11;15(11):e0009916. doi: 10.1371/journal.pntd.0009916 (PMC8664216; doi:10.1371/journal.pntd.0009916)

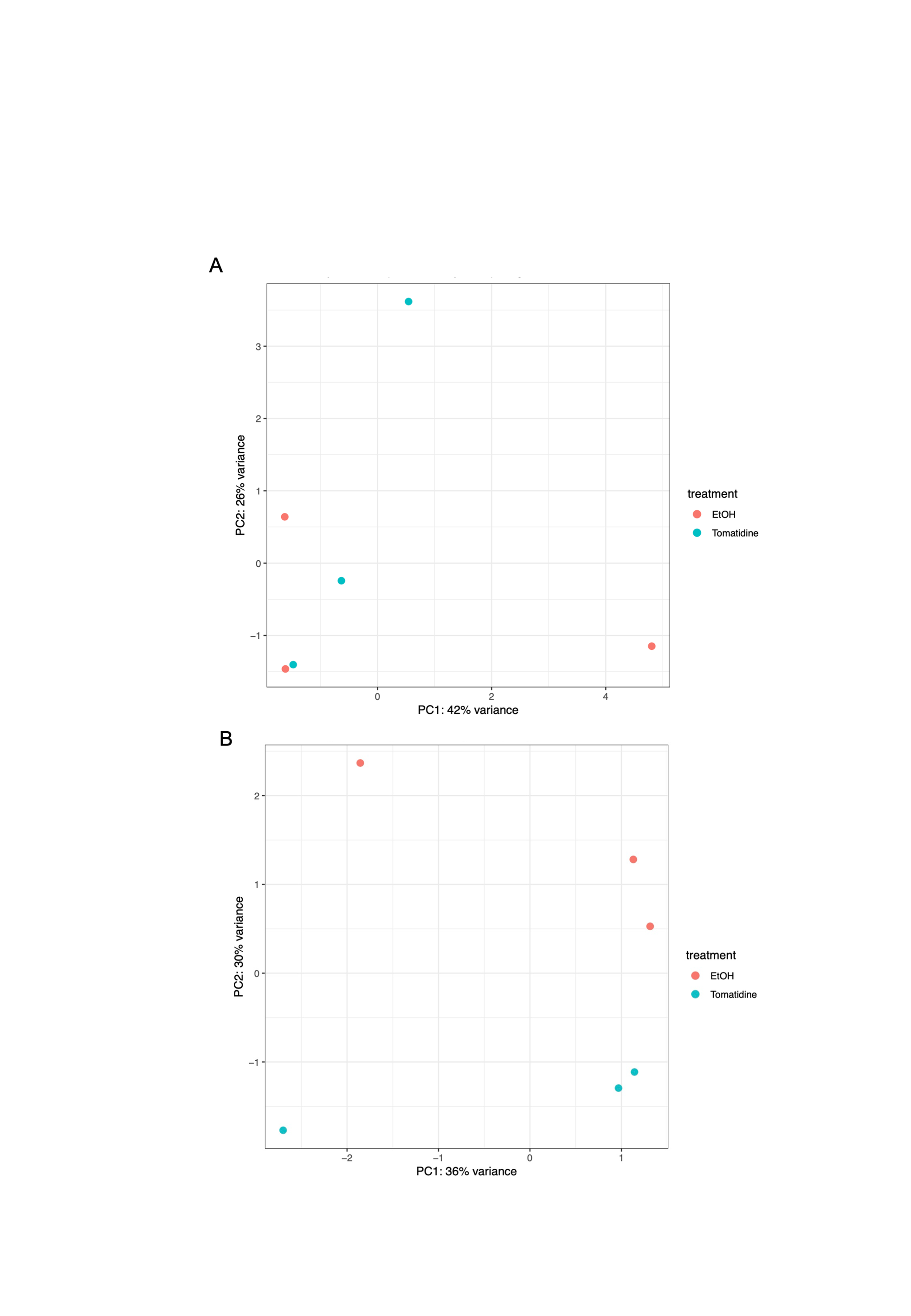

Supplement: S1 Fig — (A) Principle component analysis at 6 hpi and (B) 16 hpi. (TIF) [file pntd.0009916.s001.tif]

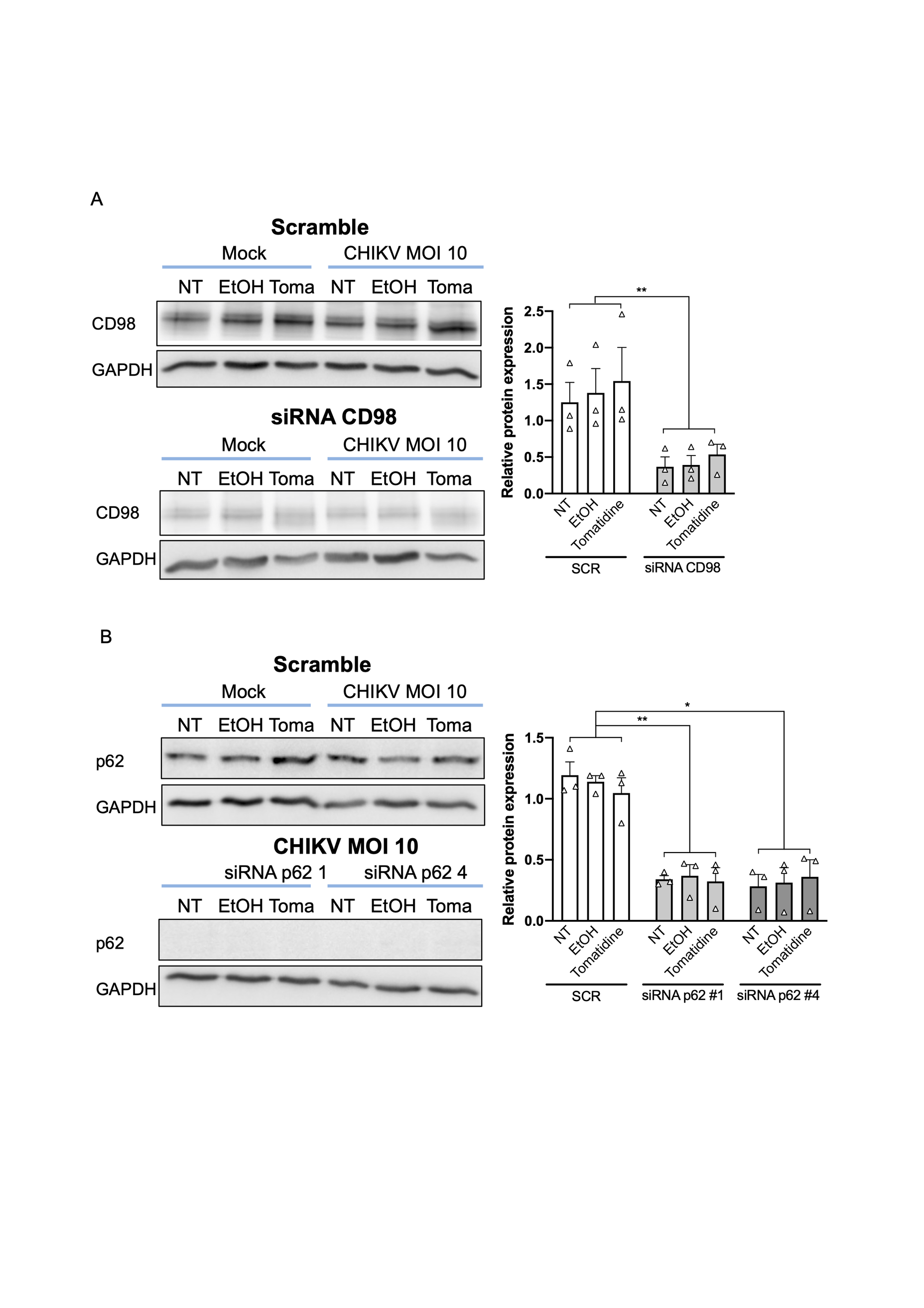

Supplement: S2 Fig — Huh7 cells were reverse-transfected with 10 nM of siRNA targeting (A) CD98 or (B) p62 or a scramble control (SCR). At 48 h post-transfection, cells were infected with CHIKV-LR at MOI 10 and treated with 10 μM tomatidine, the equivalent volume of EtOH or left non-treated. Protein was collected at 9 hpi and CD98, p62 or GAPDH as a loading control were detected via western blot. Samples were analyzed side-by-side on the same gel yet for clarity the blots were split. (A) The left panel shows the representative blots for CD98 and GAPDH. The right panel displays the band quantification normalized to GAPDH and expressed as relative protein expression compared to the mock-infected, non-treated (NT) control. (B) The left panel shows the representative blots for p62 and GAPDH. The samples were loaded on distinct gels yet the proteins bands were detected at the same time and at identical conditions. The right panel displays the band quantification normalized to GAPDH and expressed as relative protein expression compared to the mock-infected, non-treated (NT) control. For p62, two different siRNAs (siRNA #1 and #4) were used. Data are presented as mean ± SEM from three independent experiments. The statistical significance was determined using one-way ANOVA Dunnett’s multiple comparisons test. (TIF) [file pntd.0009916.s002.tif]

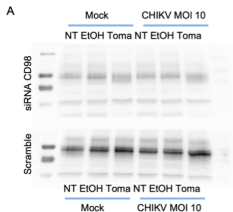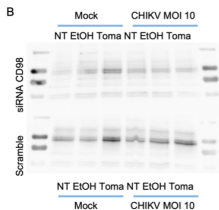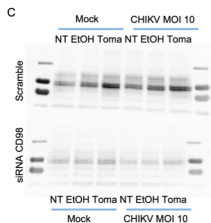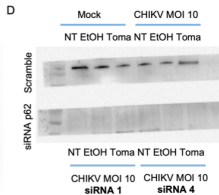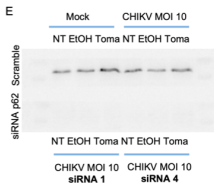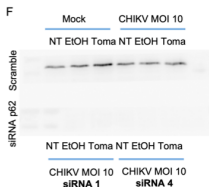

Supplement: S3 Fig — (A-C) Original western blot scans of CD98 knockdown including n = 1 (A), n = 2 (B) and n = 3 (C). (D-F) Western blot scans of p62 knockdown including n = 1 (D), n = 2 (E) and n = 3 (F). (PDF) [file pntd.0009916.s003.pdf]

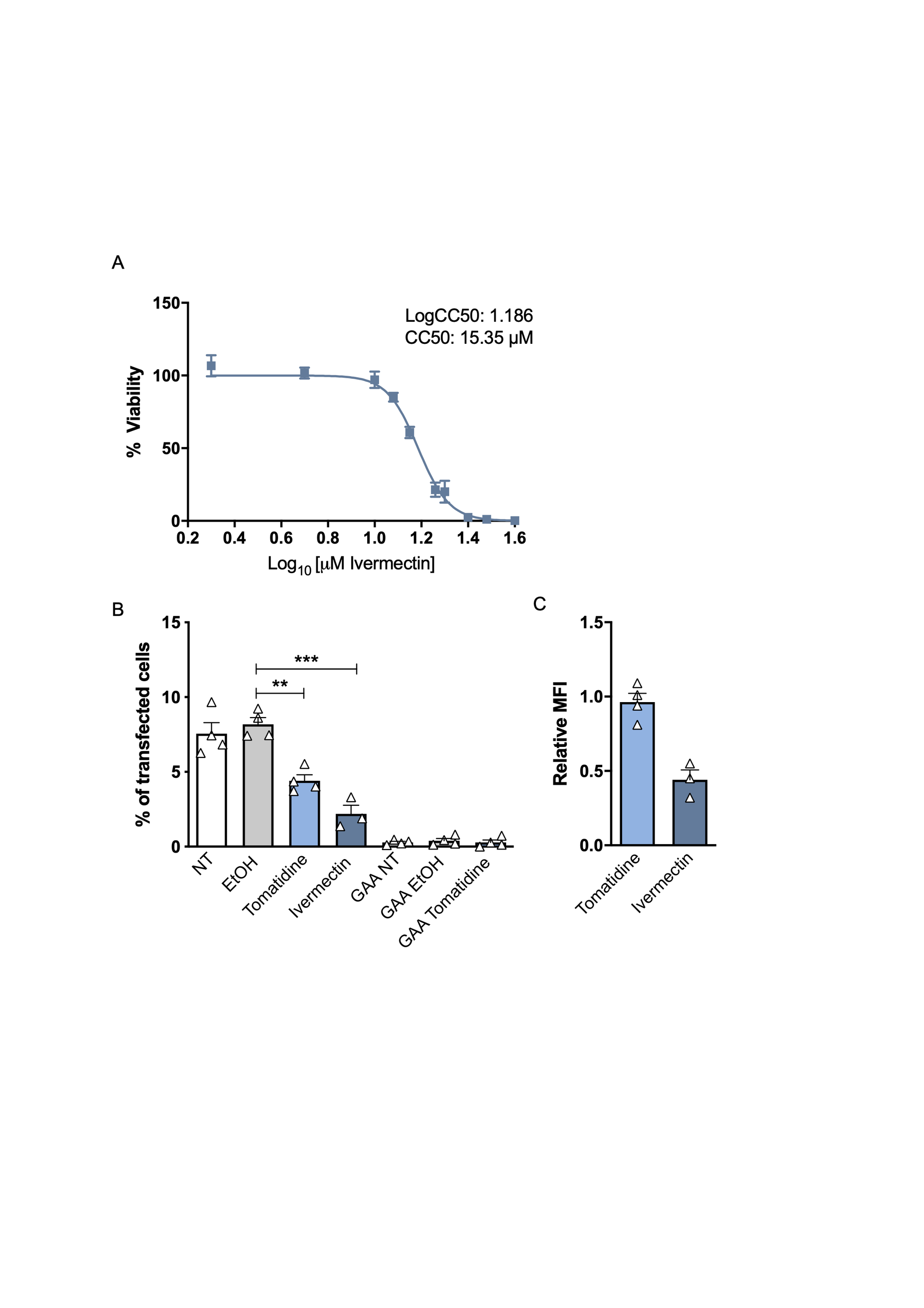

Supplement: S4 Fig — (A) Dose-response curve of the ATP level in Huh7 cells assessed by ATPLite assay in the presence of increasing ivermectin concentrations at 24 h treatment period. (B-C) Huh7 cells were transfected with CHIKV-LR trans-replicase system consisting of a template plasmid including a tomato marker and a replicase plasmid or its nonfunctional mutant (GAA). At 1 h post-transfection, cells were treated with 10 μM tomatidine, the equivalent volume of EtOH or ivermectin (7 μM or 0.8 log10) or left non-treated (NT). At 24 h post-transfection cells were collected, fixed and analyzed via flow cytometry. (B) Percentage of transfected cells detected via the fluorescent tomato marker and (C) MFI relative to the CHIKV-infected EtOH control. Data are presented as mean ± SEM from four independent experiments. The statistical significance was determined using an unpaired t-test. (TIF) [file pntd.0009916.s004.tif]

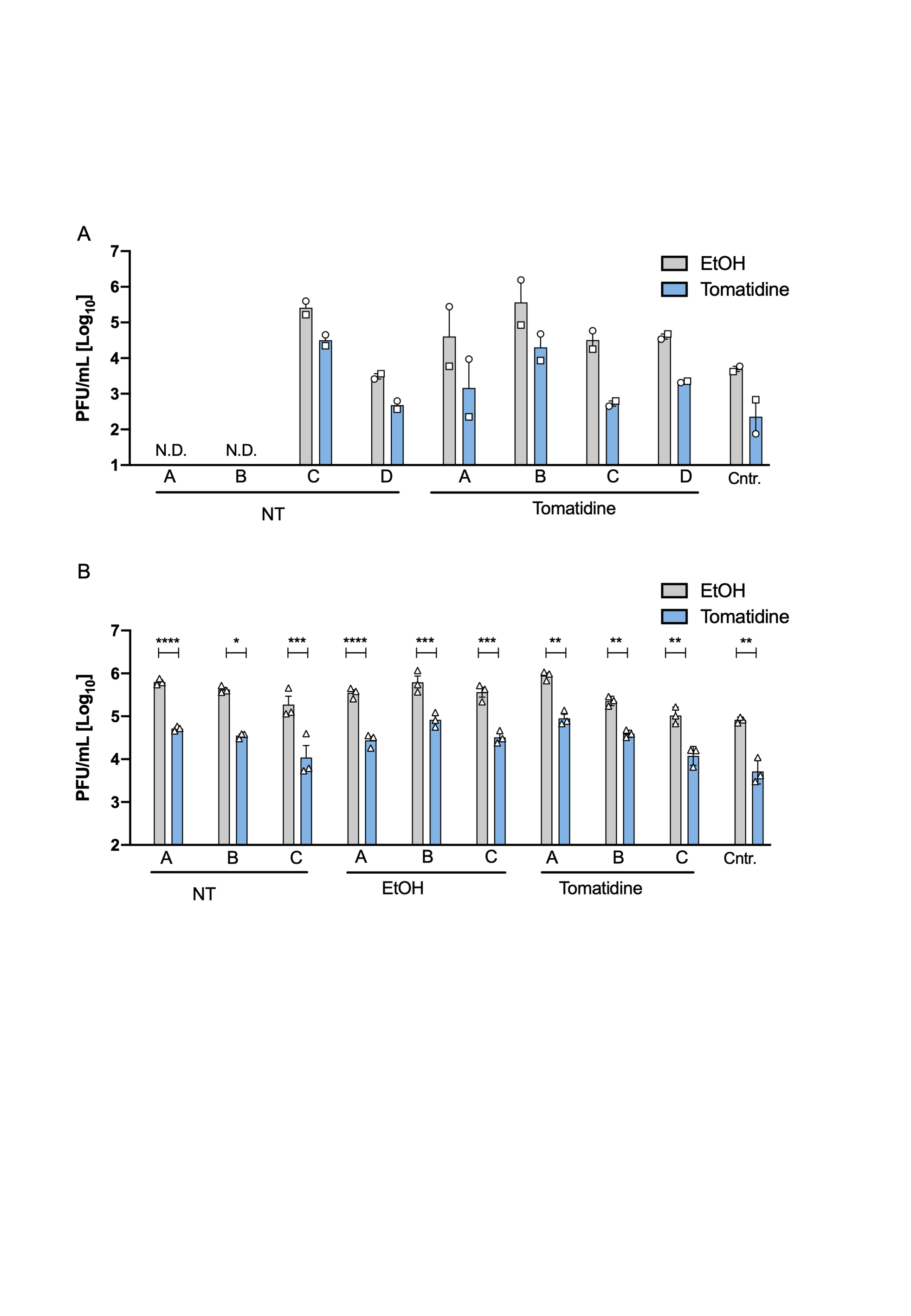

Supplement: S5 Fig — (A-B) Huh7 cells were infected with the (A) passage 15 or (B) passage 7 CHIKV samples in the presence of EtOH, tomatidine or in the absence of any compound at MOI 1. At the time of infection, cells were treated with 10 μM tomatidine or the equivalent volume of EtOH. The parental CHIKV strain was used as an internal control. Supernatants were collected at 9 hpi and infectious particle production was measured via plaque assay. Data is presented as Log10 PFU per mL. (A) A, B, C and D refer to the biological replicates. Data is presented as mean ± SEM from two independent experiments. (B) A, B and C represent the biological replicates. Data are presented as mean ± SEM from three independent experiments. The statistical significance was determined using an unpaired t-test. (TIF) [file pntd.0009916.s005.tif]
